# Survey of Sustainable Wearable Strain Sensors Enabled by Biopolymers and Conductive Organic Polymers

**DOI:** 10.3390/gels11040235

**Published:** 2025-03-24

**Authors:** Cephas Amoah, W. G. Skene

**Affiliations:** 1 Département de Chimie, Université de Montréal, Montréal, QC H2V 0B3, Canada; cephas.amoah@umontreal.ca; 2 Institut Courtois, Université de Montréal, Montréal, QC H2V 0B3, Canada

**Keywords:** resistive sensor, wearable sensors, sustainable sensors, biopolymers, PEDOT

## Abstract

The field of wearable sensors has evolved with operating devices capable of measuring biomechanics and biometrics, and detecting speech. The transduction, being the conversion of the biosignal to a measurable and quantifiable electrical signal, is governed by a conductive organic polymer. Meanwhile, the conformality of skin to the substrate is quintessential. Both the substrate and the conductive polymer must work in concert to reversibly deform with the user’s movements for motion tracking. While polydimethylsiloxane shows mechanical compliance as a sensor substrate, it is of environmental interest to replace it with sustainable and degradable alternatives. As both the bulk of the weight and area of the sensor consist of the substrate, using renewable and biodegradable materials for its preparation would be an important step toward improving the lifecycle of wearable sensors. This review highlights wearable resistive sensors that are prepared from naturally occurring polymers that are both sustainable and biodegradable. Conductive polythiophenes are also presented, as well as how they are integrated into the biopolymer for sensors showing mechanical compliance with skin. This polymer is highlighted because of its structural conformality, conductivity, and processability, ensuring it fulfils the requirements for its use in sensors without adversely affecting the overall sustainability and biodegradability of resistive sensors. Different sustainable resistive sensors are also presented, and their performance is compared to conventional sensors to illustrate the successful integration of the biosourced polymers into sensors without comprising the desired elasticity and sensitivity to movement. The current state-of-the-art in sustainable resistive sensors is presented, along with knowledge of how biopolymers from different fields can be leveraged in the rational design of the next generation of sustainable sensors that can potentially be composted after their use.

## 1. Introduction

Wearable electronics are defined as a broad class of devices that are either worn by a user or applied to the user’s skin. Their conformality with the user’s appendages without compromising the user’s quality of life makes wearable electronics interesting for a variety of applications, notably physiological sensing, biomechanical tracking, and health monitoring [1,2,3]. The advancement of the internet of things (IoTs) has created an insatiable thirst for data. This, in turn, has created a demand for sensors that can continuously provide streams of data [4,5,6]. Wearable sensors are an example such devices, providing biomechanical metrics, among other data.

Two overarching requisites of wearable sensors are as follows: (1) the capacity to translate biomechanics into a measurable electrical signal and (2) mechanical compliance with skin [7]. These are typically satisfied by a conductive film that is either deposited on or embedded in an elastomer substrate, respectively. The mechanical to electrical transduction of the conductive layer that enables the sensor can be capacitive, piezoelectric, and resistive [8,9,10,11]. Resistive sensing has spurred the field of biosensors, in part owing to the straightforward change in resistance it causes, which correlates with normal biomechanical movements, such as stretching, bending, and compression. The simple architecture of resistive sensors compared to other transducers has the further advantage of readily allowing for an evaluation of the materials used to track biomechanics [12]. Common wearable devices that are reliant on resistive strain sensors are useful for both personal health (heart rate and blood pressure) and physiological (human body movement, i.e., finger, muscles, and joint motion) monitoring, along with speech detection [13,14,15].

Although the performance of the resistive sensor is underpinned by the conductive layer, the substrate plays an equally important role in ensuring the transducer has the desired mechanical compliance. Elastomers have become the choice substrates in sensors because they can reversibly withstand the repeated stresses/strains that are encountered with biomechanical movements. Synthetic elastomers are typically used in resistive sensors, and they include polydimethylsiloxane (PDMS), poly(ethylene terephthalate) (PET), and thermoplastic polyurethanes (TPU) [16,17,18,19]. Although synthetic elastomers meet the mechanical performance requirements for use in wearable sensors, they are environmentally unsustainable. This is because they cannot be recycled after use and they are derived from non-renewable resources. As the elastomer accounts for most of the weight of a two-component strain sensor, using elastomers that are both derived from renewable sources and degradable would improve the overall sustainability of sensors. Bearing this in mind, advances in the ecological lifecycle of wearable sensors over the past five years are surveyed in this study. The use of sustainable elastomers other than hydrogels [20] in operating resistive strain sensors is the principal focus of this survey. These devices are chosen to highlight the role of renewable elastomers, because they are both well-accepted forms of wearable devices and provide established platforms for validating new materials for use in wearable electronics [21,22]. The sensor’s performance, and hence its sensitivity to biomechanical movements, are governed by the synergetic complementarity of the elastomer with the conductive layer, enabling the device’s use. For this reason, conductive polythiophenes that are structurally compliant with sustainable elastomers will also be presented. Here, sustainable wearable sensors are defined as conductive elastomers that are principally prepared from polymers originating from renewable sources such as biopolymers and transduce a given human stimulus into a direct electrical output, such as a resistance difference, when worn by a user. This contrasts with capacitive and piezoelectric sensors, which rely on an electrostatic field and alternating electroacoustic signals, respectively [23,24]. While piezoelectric and capacitive sensors offer greater sensitivity, absolute measurements, and multitouch capacity, resistive sensors excel in their simplistic architecture, and unique ability to measure resistance [25,26]. The performance of resistive sustainable sensors will be benchmarked against their environmentally deleterious counterparts. This shows the potential of renewable materials to move the field toward ecologically responsible wearable sensors with improved lifecycle management capacities. This information can ultimately be leveraged for rationally designing the next generation of high-performance sustainable sensors that can overcome the challenges faced when integrating conductive polymers with elastomers without compromising both critical conductivity and mechanical compliance.

## 2. Biopolymers for Sustainable Substrates

In the design of wearable electronics, the choice of substrate is important, as most conductors used in the sensors are not mechanically compliant with skin. Substrates used in wearable sensors must meet the following criteria. Firstly, their mechanical properties should be consistent with elastomers. This is to reversibly absorb the stresses/strains encountered when wearing the device without permanent deformation. Although there are no well-defined quantifiable metrics for the substrate, Young’s modulus ca. 10^6^ MPa and an expected elongation at break upwards of 1000% are generally acceptable [27]. These two measurable criteria are ideal for wearable applications. Secondly, the substrate must accommodate the organic conductor, either via a surface coating or through blending in the elastomeric matrix [28]. Indeed, the incorporation of the organic conductor must not compromise the mechanical properties of the substrate or alter its acceptable Young’s modulus and elongation at break. 

**Figure 1:**
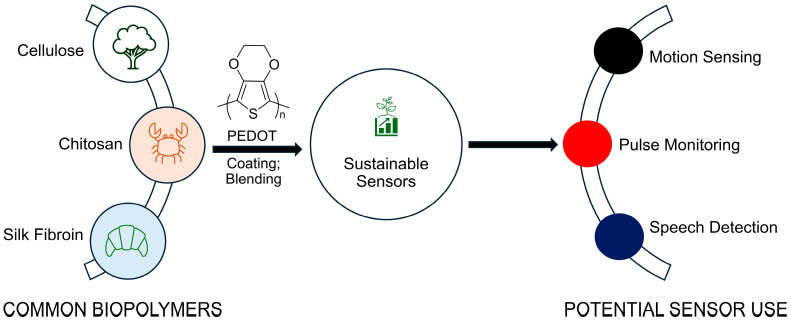
The potential use of representative biopolymers as sustainable wearable sensors that can be enabled through the conductive polymer PEDOT.

Biopolymers have emerged as suitable replacements for conventional and unsustainable substrates for the next generation of wearable electronics (Figure 1). These are derived from renewable resources that are typically biological, such as plants (cellulose), red and brown seaweed (cellulose and alginate), and from the exoskeletons of crustaceans (chitin and chitosan). The advantage of such biosourced polymers, in addition to their sustainability, is their biodegradability. The environmental footprint of stretchable substrates can be minimized via incorporating biopolymers. They also improve the lifecycle management of sensors compared to elastomers derived from non-renewable resources [29]. The collective elements of sustainable biosensors further translate into end-of-use environmental and economic advantages courtesy of their recyclability, reusability, and biodegradability, which improve the overall lifecycle management of biosensors.

These attributes are revealing new areas of underexplored research on sensors. The feedstocks used to prepare sustainable elastomers include chitosan, textiles, and cellulose and its derivatives, among others [29]. However, these biopolymers are not intrinsically elastomeric. Rather, they are brittle and do not withstand the stresses/strains of wearable devices when cast as films for use in sensors. This is due to interpolymeric hydrogen bonding. Bearing this in mind, plasticizers can successfully convert the otherwise brittle biopolymer films to stretchable and bendable substrates with mechanical compliance for use in wearable sensors (vide infra). The following sections outline three biopolymers (chitosan, cellulose, and silk fibroin) that can serve as sustainable elastomeric substrates and their use in sensors.

### 2.1. Chitosan

Chitin is a linear polysaccharide that is abundantly found in the cell walls of crustaceans and fungi [30]. It is a homopolymer consisting of *N*-acetyl glucosamine. To obtain stretchable self-standing films, chitin must be deacetylated to its chitosan counterpart. Chitosan is a random copolymer of glucosamine and *N*-acetyl glucosamine that can be prepared either chemically or enzymatically. Chitin can also be deacetylated with enzymes derived from either fungi or insects [31]. Chemically, chitosan is typically prepared by the deacetylation of chitin with alkaline solutions (NaOH and KOH). Its emerging use in sustainable strain sensors is a result of its relative abundance and straightforward modification. Deacetylation increases the amount of amines concomitant with the degree of interpolymer interactions. As a result, films cast from neat chitosan are intrinsically brittle. Their mechanical compliance for use as flexible and stretchable substrates is made possible by plasticizing with polyvinyl alcohol (PVA), glycols, and sugars [32,33,34]. Indeed, chitosan self-standing films with a tensile strength of 10–30 MPa and an elongation at break upwards of 30% are made possible through plasticization [30,35,36]. Plasticized chitosan films have been extensively used in both the agriculture and food sectors, notably for packaging [37,38]. Their non-toxicity, transparency, odorless, and resistance to moisture when chemically modified make them ideally suitable for food-contact applications [39]. Subsequently, the packaging field provides a wealth of information regarding how to tailor the mechanical properties of chitosan via chemical modification, processing, and the use of different types of plasticizers. The packaging sector has provided, and will continue to provide, pivotal understanding that can be leveraged for the rational design of chitosan substrates that meet the mechanical property requirements, including a tensile strength within the acceptable limits of ca. 10^6^ MPa, for their use in wearable sensors [30,32,33,40,41].

**Figure 2:**
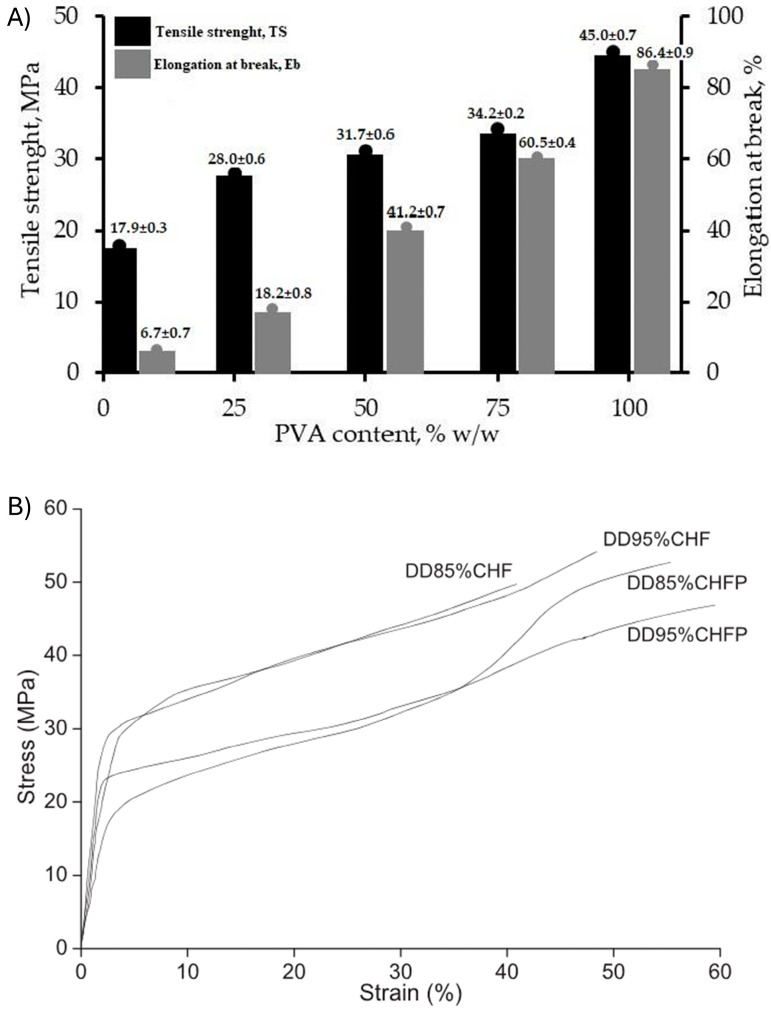
Elastic properties of chitosan films with different plasticizers. (**A**) The effect of the composition of the film on the maximum strength contingent on the chitosan/PVA weight ratio. Reproduced with Permission [36]. Copyright 2022, MDPI. (**B**) Stress–strain curves as a function of the degree of deacetylation (DD) of 85% and 95% of chitosan films with (CHFP) and without (CHF) sorbitol plasticizer. Reproduced with permission [30]. Copyright 2014, Elsevier B.V.

Transparent and flexible chitosan films have been obtained using PVA as a plasticizer at weight concentrations varying from 0 to 100% by Wardhono et al. [36]. Neat chitosan had a tensile strength and elongation at break of 17.9 ± 0.3 MPa and 6.7 ± 0.7%, respectively (Figure 2A). This contrasts with neat PVA films (without chitosan), which had a tensile strength and an elongation at break of 45.0 ± 0.7 MPa and 86.4 ± 0.9%, respectively. The mechanical properties of the chitosan films could be adjusted between the limits of the neat component by increasing the amount of PVA. Also, other plasticizers, such as glycerol and fatty acids (palmitic and stearic acids), have been used to convert the mechanical properties of pure chitosan films to meet the flexible/stretchable requirements for wearable applications [40]. The tensile strength of a neat chitosan film decreased from 39 MPa to 11 MPa when equal amounts of different plasticizers were added. Among the various plasticizers, glycerol obtained the poorest results in terms of tensile strength compared with the neat film without plasticizer. Sorbitol has also been used as a plasticizer to convert otherwise brittle films to elastomers (Figure 2B). Plasticized chitosan films had a lower tensile strength and an elongation of up to 60%. This contrasts with neat chitosan films, which had a high tensile strength and low elongation. These differences in mechanical properties were due to the different deacetylation degrees of chitosan. The advantages of using sorbitol as a plasticizer are its bioavailability and its biodegradability. Its use for plasticizing chitosan elastomers is a more sustainable option compared to the use of other plasticizers [30]. Although the elastomeric properties of plasticized chitosan make them ideal for use in solid-state sensors, their use has been limited to hydrogels [42,43].

The physical properties of chitosan that make them ideally suited for wearable sensors were illustrated by Wang et al. [42]. They synthesized polyacrylamide–chitosan–Al^3+^ hydrogels as strain sensors with skinlike properties. The sensors were transparent, highly adhesive, and self-healable owing to the coordination of aluminum with both the acrylamide and chitosan. They were also stretchable, with an elongation at break >1000%. Similarly, double-network hydrogels were prepared from a blend of chitosan, glycol, and polyacrylic acid. These also had elongations of from 870 to 1175% and they were successfully used for strain sensors, including sensors of human motion [43]. These examples illustrate how the properties of chitosan can be tailored, along with their mechanical properties, to meet the requirements for their use in wearable strain sensors.

### 2.2. Cellulose

**Figure 3:**
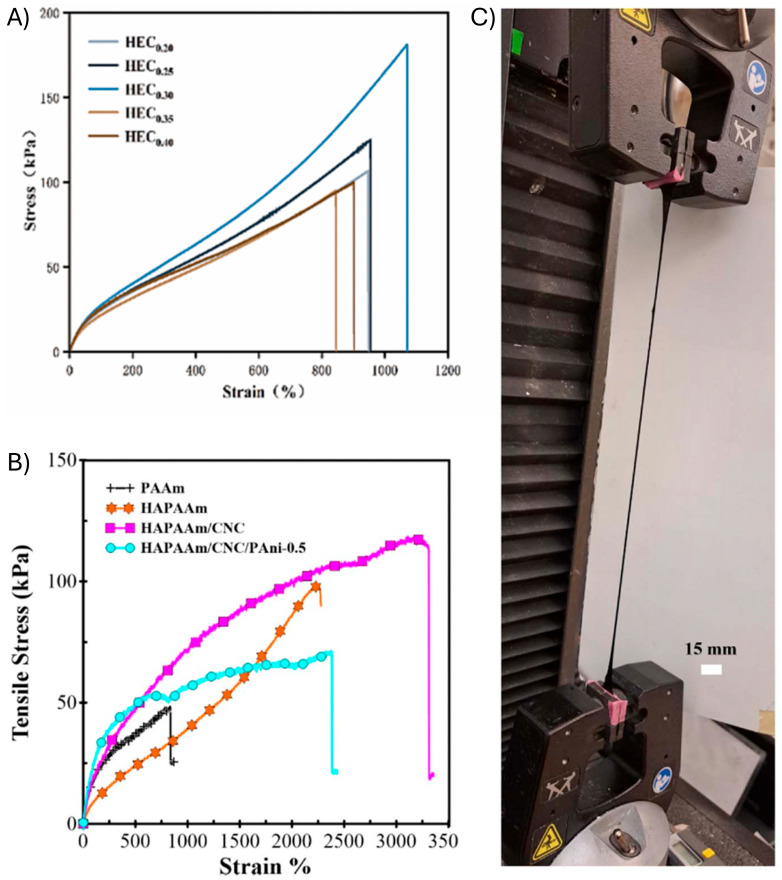
Elastic properties of cellulose hydrogels. (**A**) Stress–strain curves and the effect of the tensile strength and toughness of HPPL hydrogel with different concentrations of hydroxyethyl cellulose (HEC). Reproduced with permission [52]; copyright 2022, Springer Nature B.V. (**B**) Tensile stress–strain of PAAm, HAPAAm, HAPAAm/CNC and HAPAAm/CNC/PAni-0.5 (**C**). Photograph showing the stretchability of HAPAAm/CNC/PAni-0.5 near the ultimate strain (2400%) (polyacrylamide (PAAm); hydrophobic association polyacrylamide (HAPAAM); cellulose nanocrystals (CNC); polyaniline (PAni)). Reproduced with permission [53]. Copyright 2024, Royal Society of Chemistry (Great Britain).

Cellulose is among the most naturally abundant carbohydrate. It is available from plants, and it is extracted via a multistep delignification process. It is a biopolymer comprising multiple 1,4-β-glycosidic bonds (Figure 3) [44]. Cellulose films are becoming a popular choice for food packaging because of their non-toxicity, transparency, stretchability, vapor barrier, and antimicrobial properties [45,46,47]. Moreover, their films can withstand heat, and they can suspend solid materials. Other uses of films of cellulose derivatives and their corresponding hydrogels include in plastic cups, transparent films, and carry bags [45,46,47]. Cellulose can also be used in various forms in strain sensors. It can be used either as a nanocrystal or nanofibril [48,49]. Structurally modified cellulose structures, such as hydroxyethyl cellulose (HEC) and carboxymethyl cellulose (CMC), have also been used as substrates [50,51]. Most modified celluloses are blended to form hydrogels that are used to make strain sensors. Indeed, electrically conductive HEC hydrogels were used in strain-sensitive sensors that had an elongation greater than 1000% (Figure 3A) [52]. Increasing the HEC content increased the elongation of the hydrogel. However, the elongation stagnated with 30% w/w of HEC. In another study by Rahmani et al., hydrogels were prepared with cellulose nanocrystals. They were rendered conductive and suitable for use in wearable sensors via the incorporation of polyaniline [53]. The elongation of the hydrogel increased from 2200% to 3200% when increasing the amount of CNC in the composition (Figure 3B,C). Tuning the mechanical properties of cellulose further illustrates the importance of this biopolymer as a viable substrate for wearable sensors.

### 2.3. Silk Fibroin

**Figure 4:**
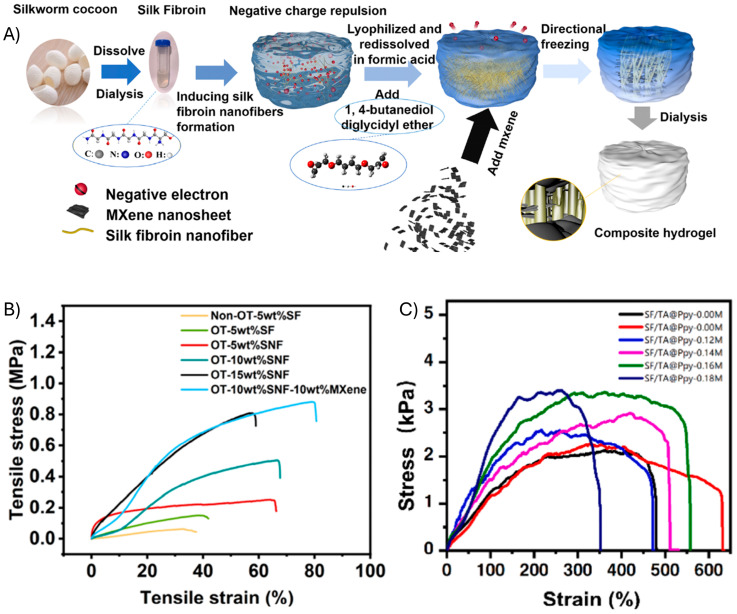
Flexible hydrogels from silk fibroin. (**A**) Schematic diagram of the preparation of the oriented MXene–silk fibroin nanofiber (MSNF) hydrogel. (**B**) Mechanical property analysis of non-oriented and oriented hydrogel samples prepared with different silk fibroin nanofiber (SNF) mass fractions. Reproduced with permission [55]. Copyright 2024, Royal Society of Chemistry (Great Britain). (**C**) Tensile stress–strain curves of silk fibroin (SF), tannic acid (TA) and polypyrrole (PPy) blend (SF/TA@PPy). Reproduced with permission [56]. Copyright 2014, Elsevier B.V.

Silk fibroins are degradable biopolymers that are obtained from silkworms. They have been used in numerous applications in recent years, ranging from biomedical devices to textiles. They are proteins composed of amino acids that are covalently cross-linked using disulfides. Silk fibroin contains antiparallel β-sheets of repeating amino acid sequences, mainly of tyrosine, serine, alanine, and glycine [54]. Numerous studies have been conducted in recent years on the use of silk fibroins and their analogs in stretchable materials. Indeed, an oriented silk fibroin nanofiber hydrogel was synthesized from a silkworm cocoon, following a multistep process to obtain both the silk fibroin and its corresponding nanofibers, which were fabricated into flexible and wearable sensors (Figure 4A) [55].

The elastic properties of the hydrogels depended on the orientation of the internal structure in terms of the interactions between the different components. Converting the silk fibroin to a nanofiber proved essential for improving the elongation to ca. 70%. The tensile strength and elastic modulus were also ideal, being 0.89 ± 0.03 MPa and 3.41 ± 0.05 MPa, respectively (Figure 4B). The elasticity of the fibers was twice that of the skin of a human forearm. The mechanical compliance of silk fibroins with skin is driving their integration into the next generation of wearable electronics. This has been spurred on by Zuo et al., who prepared a conductive hydrogel from silk fibroin by blending polypyrrole and tannic acid (SF/TA@PPy) [56]. The hydrogel was stretchable, with an elongation greater than 350% (Figure 4C). An advantage of silk fibroin is its hydrophobicity, making it suitable for strain-sensing in both air and water. Silk fibers have been incorporated into hydrogels to form freestanding films. Another advantage of silk fibers is their potential to self-heal. This is courtesy of the multiple non-covalent interpolymer interactions that reversibly cross-link the fibers. With sheer and applied stresses, the bonds dissociate and reform once the forces are no longer applied [56,57].

### 2.4. Challenges of Sustainable Substrates for Use in Strain Sensors

The sustainability and biodegradability of biopolymers such as chitosan, cellulose, and silk fibroin make them environmentally sound alternatives to conventional substrates that are derived from nonrenewable sources. The choice of biopolymers to develop a substrate for sensors is further fueled by their elongation at break, tensile strength, Young’s modulus and other key mechanical metrics that can be tailored to meet the requirements for substrates that are mechanically compliant with skin. To compete with conventional substrates, biopolymers must also have a comparable device performance to conventional strain sensors. In addition to meeting these requirements, the substrate must also be compatible with the conductive layer [58,59]. To achieve this end, substrates derived from biopolymers can be functionalized to make them more compatible with the conductive polymer. However, chemically modifying the biopolymer can alter the desired mechanical properties. Ultimately, the substrate may no longer be mechanically compliant for use as a wearable sensor [60].

Wearable sensors are further defined by their sensitivity. Sensitivity is expressed by two parameters. On one hand, it is the response time of the sensor following external stimulus. This metric is usually assigned a gauge factor (vide infra). On the other hand, sensitivity is also defined by both the upper and lower detectable range of motion. These sensitivities are underpinned by both the bulk mechanical properties of the substrate and the resistance of the conductive layer in the sensor. As such, improving the sensor sensitivity requires adjusting both the stretchability and the conductivity of the sensor. The choice of conductive material is further important because its intrinsic conductivity is not carried over when it is integrated into the device. The shelf life, including the sensor lifetime, can also be a limiting factor for a biopolymer sensor. This is owing to both the chemical and physical resistance of the substrate. Bioactivity and hydrophilicity are two potential downsides to sustainable sensors. Indeed, over time, the sensor will degrade naturally due to its absorbance of ambient and user moisture. This will decrease the sensors’ sensitivity over time and accelerate degradation of the biopolymer before its true end-of-life [61,62]. Studies that can fill the voids in the knowledge of how to improve the long-term stability will be beneficial to advancing the field. This knowledge will also be key to ensuring that substrates derived from biopolymers can outperform sensors derived from nonrenewable resources in addition to providing environmental benefits.

## 3. Organic Conductive Polymers in Sustainable Wearable Sensors

### 3.1. Polythiophene Sustainable Strain Sensors

**Figure 5:**
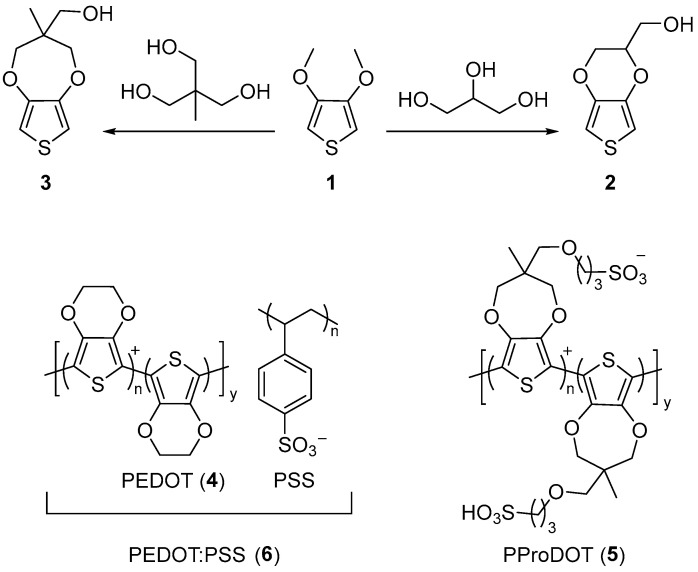
Reaction scheme for the synthesis of hydroxyl derivatives of ethylenedioxythiophene (**2**) and propylenedioxythiophene (**3**) from 3,4-dimethoxythiophene (**1**), along with their representative conductive polymer counterparts (**4**–**6**).

Numerous materials have been investigated as resistance transducers for biomechanical sensors. These include metal oxides, nanoparticles, carbon materials (nanotubes and graphene), ionic conductive hydrogels, and intrinsically conductive polymers [63,64,65,66,67]. Conducting composites consisting of mixed conducting materials have also been explored for enabling strain sensors [16]. Despite the sensitivity of these conductive materials, and their compatibility with skin-like substrates, which positively impact their use as resistive transducers, new materials are required to meet the high-performance requirements of strain sensors while satisfying the environmental challenges. It is important that the next generation of materials possess improved processability with substrates, enhanced conductivity, and improved elastic properties. They must also have a reduced environmental footprint. The sensors must further withstand the repeated stresses/strains that are encountered during regular sensor use without permanently deforming, along with having an extended shelf life. Conductive polythiophenes are ideal candidates to meet these criteria while filling the performance void. This is in part owing to their conductivity, which can be tailored via structural modifications. Modifying the lateral constitutional components of polythiophenes further provides the means to tailor physical properties other than their conductivity and make them chemically compliant with the substrates that are used for wearable devices. The collective chemical, mechanical, and electrical modulation of polythiophenes sets them apart from other conductors used in biomechanical strain sensors [68]. Indeed, the high conductivity of doped poly(ethylenedioxythiophene) (PEDOT) (**4**, Figure 5), which is upwards of 4000 S/cm [69], makes it the polymer of choice for enabling a wide range of electronic devices [68,70,71,72]. Its structurally similar counterpart poly(propylenedioxythiophene) (PProDOT) (**5**) also plays an important role in electronic devices. For example, the contrasting colors of doped and undoped PProDOT have been exploited for electrochromic applications [70]. A virtue of both PEDOT and PProDOT is that their pendant substituents can be modified without affecting the desired conductivity for their use in sensors, which they possess when they are doped [68,70]. Indeed, the lateral components of these conductive polymers can be modified without compromising the conjugated framework. The processing of conductive **4** and **5** for their integration into otherwise hydrophilic substrates can be tailored for blending without phase separation by tuning the pendant substituents. This is possible when using the hydroxyl derivatives of EDOT (**2**) and ProDOT (**3**). These can be prepared from the common reactant, 3,4-dimethoxythiophene (**1**) via acid-catalyzed alkylation with 1,1,1-propanetriol and tris(hydroxymethyl)ethane, respectively (Figure 5) [68,72].

Although EDOT and ProDOT are not derived from sustainable sources, their incorporation into sensors does not overshadow the overall sustainability of the device. This is because reduced amounts (<20 wt.%) of the conductive polymer were required to enable the device compared to the bulk of the components used for sensor fabrication. This is also the case with other conductive polymers, such as PEDOT:PSS and polyaniline [73,74]. Conductive polymer loadings can even be lowered so that they account for <5 wt.% of the sensor [75]. The environmental impact of PEDOT and PProDOT is further minimized by using earth-abundant catalysts (Fe^3+^ salts) and environmentally innocuous solvents, such as water, for their preparation [71,76]. This contrasts with their metal oxide counterparts, which are derived from rare earth metals and whose mining has negative environmental effects. Incorporating conductive thiophenes, such as **4** and **5**, into sustainable devices, is not expected to prevent the decomposition of the device at the end of its life due to the known biological compatibility of conductive polymers [77].

PEDOT:PSS (**6**) has enabled sustainable strain sensors to be developed. This is owing to its intrinsic conductivity, which is suited for use in resistance sensors. Of importance is the resistance of PEDOT:PSS, which varies with different stimuli. Its high conductivity also ensures that the sensors it enables have high sensitivity. **6** also meets the requirements of green chemistry because it is dispersed in innocuous water. The fully water-soluble counterparts of **6** have not been used in sustainable strain sensors despite their compatible processability with sustainable substrates [78,79]. While the field is in its infancy, most sustainable sensors incorporating PEDOT have focused exclusively on hydrogels, films, and textiles.

The majority of strain sensors presented are human motion sensors. These track microscopic movements, such as vibrations in speech and arterial pulse, among others. They are fabricated by blending the conducting polymer with the sustainable material to form a hydrogel. Alternatively, the conducting polymer is coated on the substrate using various methods, including spray- and spin-coating (Table 1). The conductive polymer is either absorbed on the surface or chemically bonded to the surface upon deposition. These interactions improve the polymer’s adhesion to the substrate, ensuring it does not delaminate during device operation. Hydroxyethyl cellulose, cotton fabrics, chitosan, and nanocellulose (crystals and fibrils) are substrates that have been used in strain sensors with PEDOT:PSS.

Although PEDOT:PSS intrinsically has a high conductivity, its conductivity decreases when the polymer is integrated into a sensor. The conductivity is nonetheless suitable for enabling the sensor. This is summarized in Table 1, which shows that the conductivity of the PEDOT:PSS is contingent on the biopolymer used to fabricate the sensor. Mixing PEDOT:PSS with the biopolymer through means such as coating or blending affects the conducting polymer–biopolymer interactions and, in turn, the overall conductivity/sheet resistance. The lower conductivity is 0.017 S/m, and this can be increased to upwards of 140 S/m depending on the substrate. Undesired low conductivities can be increased to desired values by exchanging the PSS dopant with other molecular dopants after coating the substrate with PEDOT:PSS [80]. Mineral acids and co-solvents have successfully been used as dopants to enhance the PEDOT:PSS conductivity to levels that are ideal for enabling sensors [81,82]. The dopant plays an important role in modulating the conductivity, and hence both the overall sensitivity and the limit of detection of the strain sensor.

**Table 1:** Summary data of sustainable resistance-type strain sensors studied.

Sensor Composition ^1^	Integration of PEDOT	Sustainable Component/Substrate	Conductivity	Applications Studied	Ref.
PAVK-PEDOT:PSS-PANS bilayer hydrogel	Blending and Spray-Coating	Carboxylated cellulose nano whiskers/carboxymethyl chitosan	1.76 S/m	-	[83]
CNC-PEDOT:PSS/PVA hydrogel	Blending	Cellulose nanocrystals	4.73 S/m	Human motion (wrist, finger, knee, and neck motion)	[84]
CMC-PEDOT:PSS composite film	Blending	Carboxymethyl cellulose	5.5 × 10^2^ Ω/sq.	Human motion (finger, throat, skin-wrinkling) and speech	[51]
PEDOT/PSS/CNF aerogel	Blending	Cellulose nanofibrils	140 ± 30 S/m	-	[85]
PVA/Gly-CNC/PVP/PEDOT films	Blending	Cellulose nanocrystals	0.017 ± 0.001 S/m	Human motion (finger, palm, elbow, neck, pulse, and smile)	[86]
PEDOT/GNP Fabric	Spray-coating	Cotton fabric	25 Ω/sq.	Human motion (finger)	[87]
PEDOT/MWCNT Fabric	Spray-coating and in situ polymerization (adsorption)	Polyester–latex mesh fabric	121 S/m	Human motion (knee, finger and wrist)	[88]
PEDOT:PSS/HEC film	Spin coating	Hydroxyethyl cellulose film	581 Ω/sq.	Human motion (skin wrinkling, touch, finger, wrist, breathing, and walking)	[89]

^1^ PAVK: polyacrylamide, polyvinyl pyrrolidone, and potassium chloride. PANS: polyacrylamide, poly(*N*-methylol) acrylamide, carboxymethyl chitosan, and calcium chloride. CNC: cellulose nanocrystals. CNF: cellulose nanofibers. PVA: polyvinyl alcohol. GNP: graphene nanoparticles. MWCNT: multiwalled carbon nanotubes. HEC: hydroxyethyl cellulose.

### 3.2. Mechanical Properties of PEDOT Sustainable Sensors

The overarching mechanical requirements of wearable sensors are both flexibility and stretchability. These are quantified according to the elongation at break (E_b_), tensile strength, percentage strain, and elastic modulus [16]. These mechanical properties are defined accordingly. The elongation is the maximum strain with which a material can be stretched before it is permanently deformed. The material ruptures when it is stretched beyond its maximum elongation. Tensile strength is the maximum stress that can be withstood before the material ruptures with an applied force. The strain percentage is the ratio of the extension of the material under stress compared to its initial length. The elastic modulus compares the stress applied to the strain. Both the elastic modulus and the tensile strength provide key information about a material’s flexibility and stretchability. For example, an elastomer is defined by a similarly low elastic modulus and tensile strength. A high tensile strength is characteristic of a rigid and inflexible material.

**Figure 6:**
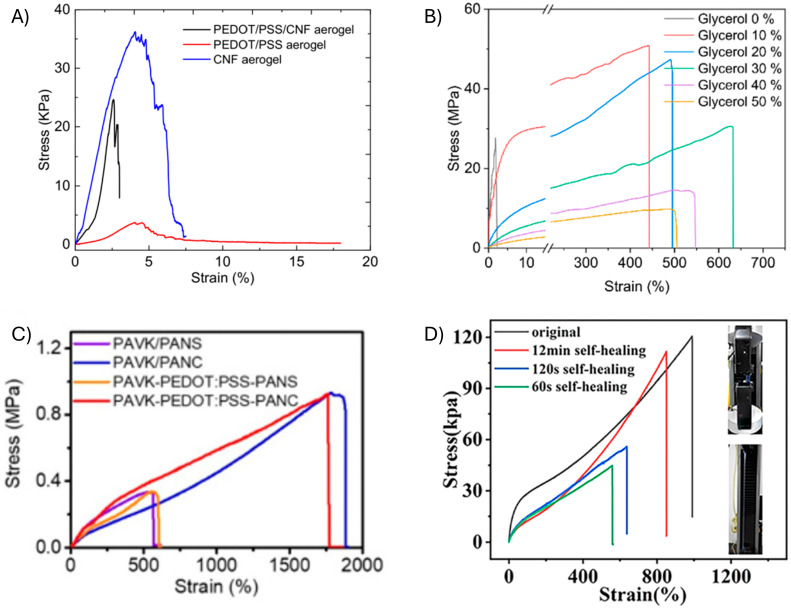
Mechanical properties of doped PEDOT in sustainable strain sensors fabricated by blending. (**A**) Stress–strain curves of cellulose nanofibers (CNF), PEDOT/PSS, and PEDOT/PSS/CNF50 aerogels. Reproduced with permission [85]. Copyright 2018, American Chemical Society. (**B**) Representative stress–strain curves of PVA/Gly-CNC/PVP/PEDOT with different glycerol weight ratios to glycerol (Gly) and PVA. Reproduced with permission [86]. Copyright 2021, American Chemical Society. (**C**) Stress–strain curves of PAVK/PANS, PAVK/PAN-CCNWs, and PAVK-PEDOT:PSS-PANC bilayer hydrogels. Reproduced with permission. Polyacrylamide (PAAm); poly(vinyl pyrrolidone) (PVP); poly(*N*-methylol acrylamide) (PNMA); carboxylated cellulose nano-whiskers (C-CNWs); carboxymethyl chitosan (CMCS); calcium chloride (CaCl_2_); potassium chloride (KCl). Hydrogel composition: PAAm/PVP-KCl (PAVK) and PAAm/PANC-CNWs (PANC) [83]. Copyright 2022, MDPI. (**D**) Tensile stress–strain curves of CNC-PEDOT:PSS/PVA hydrogels before stretching and different healing times after stretching (insets show the digital images of the hydrogel during tensile tests). Reproduced with permission [84]. Copyright 2023, American Chemical Society.

The mechanical properties of the sensor are underpinned by both the substrate and the conductive polymer. Unfortunately, most conducting polymers are not completely mechanically compliant with the flexible/stretchable substrate. As a result, their incorporation affects the overall mechanical performance of the sensor, even at low concentrations. For example, Hsiesh et al. demonstrated that the elongation at break of an aerogel sensor prepared from flexible cellulose nanofibrils was reduced to <20% strain when PEDOT:PSS was incorporated [85]. The conductive polymer did not improve the elasticity of the sensor (Figure 6A). The desired elongation at break can also be tailored by varying the plasticizer/conductive polymer ratio in a composite. This adjusts the free volume within the matrix by tailoring the plasticizer–matrix interchain interactions in the composite. Also, by modulating the amount of glycerol in a cellulose nanocrystal (Gly-CNC) polyvinyl alcohol (PVA)/poly(vinyl pyrrolidinone) (PVP)/PEDOT:PSS sensor film, the elongation at break was extended to between 400% and 700%. This contrasts with the <10% strain of the composite in the absence of a plasticizer (Figure 6B) [86].

The supramolecular structure of the biopolymer also affects the mechanical properties of the substrate. For example, nanocellulose improves the distribution and the alignment of the components in the sensor matrix. This is owing to its high surface area, which increases the number of interactions with the various components. Ultimately, composites are more readily formed with nanocellulose than its linear counterpart. Indeed, Peng et al. showed that the structural form of two sustainable materials, carboxylated cellulose nano whiskers (C-CNWs) and carboxymethyl chitosan (CMC), affected the mechanical properties of a bilayer polyacrylamide (PAAm)/polyvinyl pyrrolidone (PVP) hydrogel [83]. The elongation at break of both sensors was >500% strain. However, the elongation at break of the C-CNW sensor was three-fold higher than its CMC counterpart sensor (Figure 6C).

An often overlooked trait that can shape the future of wearable sensors is self-healing. This provides the advantage of not requiring the mechanical properties to be modified at the expense of lowering the conductive polymer content, which would otherwise worsen the device performance. Instead, the tensile strength and elongation at break can be lowered to match those of the skin. With self-healing, the otherwise permanent deformation of a sensor with applied stresses/strains, which would render it useless, can be repaired. Indeed, the sensor can be restored to its pristine state over time. The time required for the substrate to return to its original state after the application of these forces is contingent on many factors, including the type of biopolymer, its glass transition temperature (T_g_), and the type and quantity of the additives [90,91]. Rapid healing is desirable compared to slow repairing times. This would make the sensor usable immediately after an applied deformation force.

Reversible bonds can be leveraged to create self-healing sensors. For example, cellulose nanocrystals with a high surface area were blended with polyethyleneimine, PEDOT:PSS, and PVA, and cross-linked with borax. The PVA played two roles in the sensor: it served as a plasticizer and allowed for reversible bond formation with a borax cross-linker. The sensor was self-healing owing to the weak but reversible bonds between borax and PVA, which are broken during elongation at break. The self-healing ability was a result of the reversible formation of hydrogen bonds with PVA (Figure 6D) [84].

**Figure 7:**
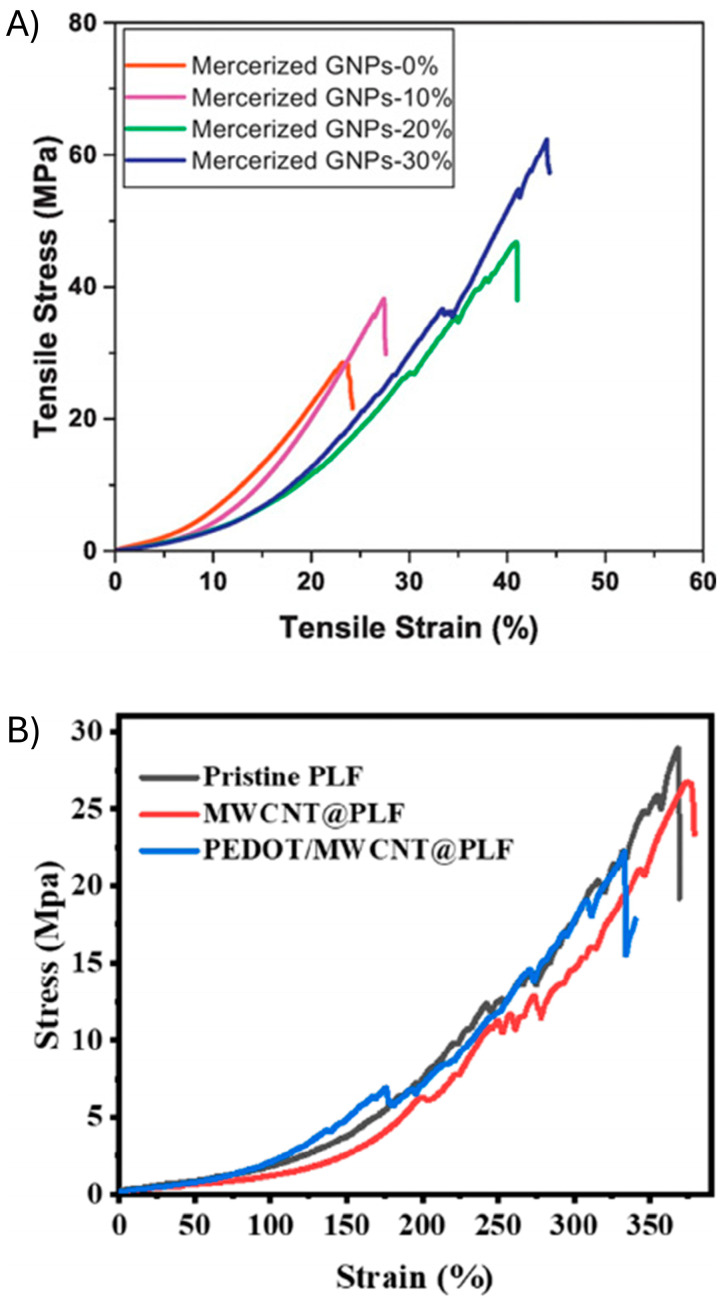
Mechanical properties of sustainable strain sensors obtained through surface-coating with doped PEDOT. (**A**) Stress–strain curves of PEDOT:PSS-coated cotton fabrics with the mercerization of different amounts of graphene nanoparticles (GNPs) (0%, 10%, 20% and 30%). Reproduced with permission [87]. Copyright 2017, Elsevier B.V. (**B**) Stress–strain curves of pristine PLF, MWCNT@PLF, and PEDOT/MWCNT@PLF. Reproduced with permission [88]. Copyright 2024, Springer Nature B.V.

The sensor architecture also affects the mechanical properties of the sustainable sensor. Two principal architectures are used for sensors: (1) blending the components to make a composite or (2) using two discrete layers to form a conductive coating on a substrate. Blending relies on the use of bulk intermolecular interactions to make a homogeneous composite. Surface coating, on the other hand, provides a thin film of the conductive polymer that is deposited on the surface through either spin- or spray-coating. This approach takes advantage of the attractive surface forces between the substrate and the conductive polymer. It is also reliant on the hydrophobic compatibility of the two layers. For example, upon elongation and sheering, a hydrophilic substrate and a hydrophobic conductive polymer will separate at their interface. This will result in sensor failure. Such incompatible surfaces are more suitable for low-strain micromovement applications, such as speech detection. Indeed, cotton fabrics mercerized with PEDOT:PSS showed >20% elongation, making them ideal for human-motion sensing (Figure 7A) [87]. The elongation could be improved through the addition of graphene nanoplatelet. This additionally increases the conductivity, and, in turn, the sensor’s sensitivity. A coating method was also used to make a textile sensor that had an elongation above 300% (Figure 7B) [88]. The sensor was fabricated by polymerizing EDOT directly on a polyester–latex–mesh fabric (PLF) that was previously coated with multiwalled carbon nanotubes (MWCNT). While the surface-coating method has the advantage of being rapid, it is not ideal for highly stretchable sensors. Blending the conductive polymer in the stretchable substrate is better-suited for high-elongation applications. This requires judiciously selecting components that have a high surface area to ensure matrix homogeneity and, ultimately, self-healing.

### 3.3. Sustainable vs. PDMS Sensors

Polydimethylsiloxane substrates have been the go-to option as the substrate for numerous flexible and stretchable electronics, including strain sensors [92,93,94,95]. Strain sensors with PDMS have either been blended or coated with the conductive polymer. Table 2 compares both the type of strain sensor and its conductivity, which is contingent on the use of conventional PDMS and a biopolymer substrate. While the use of PDMS in stretchable electronics has been successful, incorporating conductive PEDOT remains challenging because of its processing limitations. This is partly owing to PEDOT:PSS having an aqueous dispersion, making it immiscible with hydrophobic PDMS. Surfactants such as Zonyl and Tween are required to mix the two otherwise immiscible phases and make a homogeneous sensor [22,96]. In contrast, sustainable substrates are miscible with doped PEDOT because of their common hydrophilicity. The compatibility of the substrate and doped PEDOT is evident from the mechanical properties summarized in Table 2. The range of stretching is consistent for PEDOT-coated sensors regardless of the type of substrate. Bearing this in mind, and taking the sustainability and recyclability of biopolymer substrates into account, sustainable sensors are advantageous [51,97]. This is epitomized by the blended PEDOT:PSS/PDMS strain sensors, whose elongation at break is 10-fold lower than their sustainable counterparts [83,96]. This is due to the reduced interactions between PDMS and PEDOT:PSS. It is evident that sustainable sensors have a similar device performance to their PDMS counterparts while having an improved mechanical performance and environmental benefits.

## 4. Sustainable PEDOT Strain Sensors

### 4.1. Sensor Performance Criteria

Similar to most sensors, sustainable strain sensors have been used for a number of applications, such as motion, touch, and speech detection, to name but a few [25,98,99]. The sensor is governed by the electromechanical response of the substrate. This is the conductivity change contingent on the applied stress/strain. The sensitivity of the sensor is defined as the smallest movement that can be detected. Meanwhile, the response is defined as how rapidly the sensor responds to the external stimulus. The sensitivity is determined via the gauge factor. This is the ratio of the change in resistance to the change in strain. Although there is no commonly accepted gauge factor that defines an ideal sensor, sensitivity >1 is arbitrarily characterized as a sensitive sensor, whereas gauge factors >20 are determined to be highly sensitive sensors [17,83,96,100]. Another property that limits the application of strain sensors is their elastic hysteresis. This is how the sensor withstands multiple stress–strain cycles without being permanently deformed. Both the gauge factor and the hysteresis define the ultimate use of the sensor for either macro- or micromotion detection.

### 4.2. Macromovement Sensors

**Figure 8:**
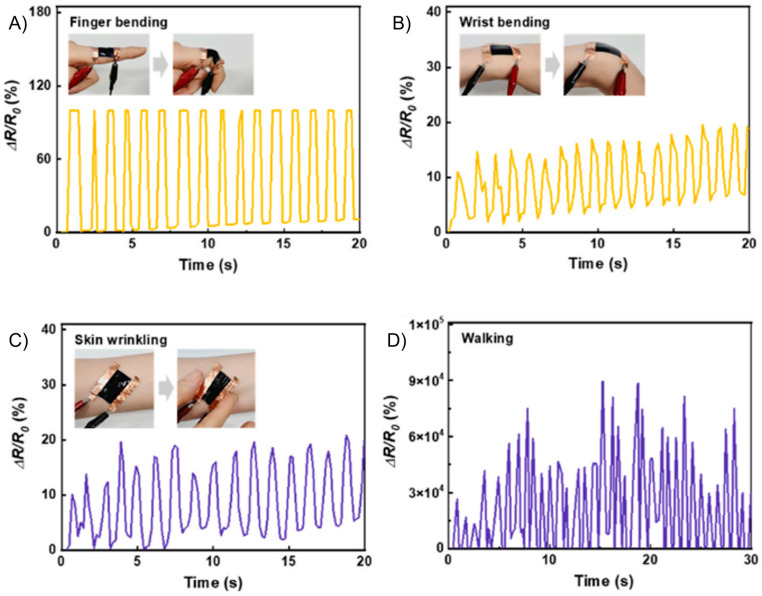
Macromovement detection with sustainable strain sensors with doped PEDOT. Response curves of strain sensors prepared from PEDOT:PSS films monitoring human movement: (**A**) finger-bending, (**B**) wrist-bending, (**C**) skin-wrinkling, (**D**) and walking. Reproduced with permission [89]. Copyright 2022, Elsevier B.V.

Like most conventional strain sensors that measure motion accurately owing to their low tensile strength (ca. 10^6^ MPa) and hysteresis [93,101,102], sustainable sensors are also used to track macromotions. These are large amplitudes of strain/stress that occur with the movement of appendages (bend, stretch, twist), muscle contraction/relaxation, skin-wrinkling, and eye-blinking [51,84,86]. A high sensitivity is not required for macromovement sensors. Rather, a large degree of motion must be detected. Sensor hysteresis is equally important, as the sensor must consistently and reliably change its resistance over the full range of motion. Han et al. developed a sustainable strain sensor by depositing PEDOT:PSS on a hydroxyethyl cellulose film. The sensor maintained its hysteresis over 100 cycles of elastic deformation at a strain of 30%. Indeed, the sensor maintained a consistent change in resistance during 500 bending/relaxing cycles [89]. The sensor tracked both finger- and wrist-bending, skin-wrinkling, and walking motions (Figure 8). While macromovement can readily be tracked, the challenge is to consistently measure the change in resistance after numerous stress–strain cycles. This is problematic because of the internal energy losses in the sensor that occur during multiple stretch/release cycles. This is compounded by the non-uniform motion of the sensor, resulting in inconsistent resistances for the same range of motion. While these inconsistencies affect neither the sensor’s performance nor its sensitivity, they affect both the dynamic range that can be detected and the absolute resistance.

### 4.3. Micromovement/Vibration Sensors

**Figure 9:**
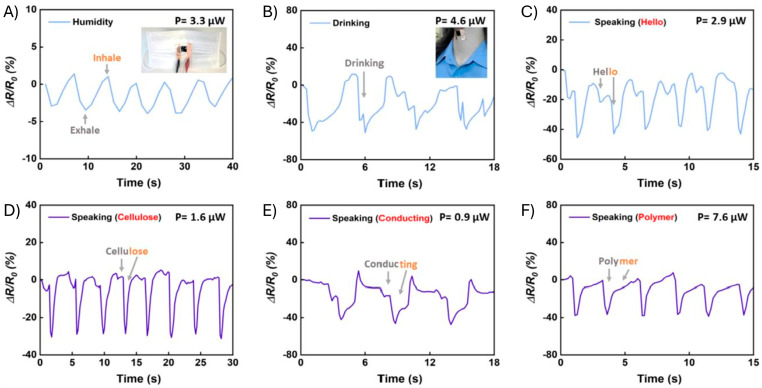
Micromovement/vibrational application of a sustainable polythiophene-based strain sensor. Response curves of on-skin sensors based on CMC-PEDOT:PSS film for respiratory humidity (**A**), drinking (**B**), and speaking the words hello (**C**), cellulose (**D**), conducting (**E**), and polymer (**F**). Reproduced with permission [51]. Copyright 2022, MDPI.

Micromovement sensing is the detection of vibrations. This class of resistive strain sensors is attracting increasing interest owing to its potential use in both speech detection/recognition and healthcare monitoring, especially for pulse detection [6]. Micromovements are more challenging to detect compared to their macromovement counterparts. This type of sensor must have a high sensitivity with a gauge factor of preferably >20. Also, it must be capable of sensing small changes in resistance that correspond to the equally small vibrational changes that are to be measured. As a result, a sensor with a low limit of detection for sensing changes in stress/strain is ideal for a micromovement sensor. The sensor must also consistently produce identical changes in resistance over time. This is the major challenge for this class of sensor. Common vibrational motions that have been studied with sustainable sensors include drinking/swallowing, pulse, and speech (speaking) (Figure 9) [51]. While the field continues to evolve, with recent studies highlighting the achievement of real-time micromovement detection, little is known about the limit of detection, especially when using conducting polythiophene strain sensors [103,104,105]. Integrating machine learning with the sensor is ideal for generating meaningful and consistent data. This will be useful for both anonymizing and normalizing user-dependent data for the universal detection of speech from fluctuations in resistance amplitudes via Savitzky–Golay deconvolution [106]. Deciphering phonation and speech independently of the user would be useful for computer-interfacing and controlling robotic devices via spoken commands.

## 5. Perspectives and Outlook

Sustainable strain sensors are an important step towards improving both the environment and the quality of life of users. While great strides have been taken towards improving the mechanical compliance and sensitivity of strain sensors that are derived principally from renewable and biodegradable feedstocks, many voids remain in the knowledge required for them to surpass the performance of their unsustainable counterparts. Among the many gaps in knowledge are the choice and the form of the sustainable substrate. These underpin the mechanical properties of the substrate. These key parameters also affect the dispersion of the conductive component in the stretchable/flexible matrix. Three biopolymers (chitosan, cellulose, and silk fibroin) have been instrumental in developing sustainable substrates that meet the key mechanical requirements for their use in sensors. Their formulation with conductive polythiophene to meet the electrical performance requisites concomitant with the success of biopolymers in hydrogels can be leveraged to expand sustainable substrates for sensors beyond cellulose, chitosan, and silk fibroin. Meanwhile, the processability and compatibility of conducting polymers such as doped PEDOT are important for advancing sustainable sensors. Conductive polymer functionalization will be critical to make conductive polymers miscible in water and to ensure homogeneous processing with biopolymers. Toward this end, water-soluble conductive polythiophenes that are self-doped will become pivotal for enabling sensors. Future directions of the field will include making sensors transparent and fabricating sensors exclusively from water. The intrinsic conductivity of sustainable sensors can further be leveraged to include additional modes of detection, such as amperometry, colorimetry, and fluorimetry. These transductions, coupled with selective biological signaling, such as unique signaling redox processes, antigens, and aptamers to name but a few, open the possibility of enhancing the resistive sensors so that they can be used as selective biosensors. Selectivity can ultimately be designed into the biosensor through integrating reporters for targeted detection. The field of hydrogels has a rich history of using biopolymers because of their stretchability/flexibility. This is a fertile ground to acquire vital knowledge that can be leveraged for the rational design of self-standing dehydrated films that meet the requirements for their use in sensors. The food packaging sector is also a prime field that can provide a wealth of information for integrating previously unused biopolymers into resistive sensors [107]. This is spurred on by the mutually common mechanical properties of the two disciplines, which include tensile strength, conformality, and stretchability. Emerging biopolymers from the food packaging and biosensor sectors that are expected to positively impact strain sensors include alginate, starch, pullulan, and gelatin. Additionally, micromovement sensors are poised to benefit from the use of artificial intelligence for speech recognition and expand into soft robotics and computer interfacing.

## Figures and Tables

**Table 2:** Comparison of the performance of PDMS resistance strain sensors and their sustainable counterparts.

Sensor Composition ^1^	Elastomer/PEDOT Integration	Elastic Modulus	Elongation at Break (E_b_)	Tensile Strength	Ref.
PEDOT:PSS/Zonyl/PDMS	PDMS sensor fabricated by blending	~6.8 MPa	5–15%	5–25 MPa	[22]
PEDOT-PSS/PDMS	PDMS sensor fabricated by coating/printing	-	150%	1.43 ± 0.03% MPa	[97]
PEDOT:PSS/Tween/PDMS	PDMS sensor fabricated by blending	-	75%	0.8 MPa	[96]
PAVK-PEDOT:PSS-PANS/PANC bilayered hydrogel	Sustainable sensor fabricated by blending	70 ± 8 kPa	1800 ± 200%	0.92 ± 0.08 MPa	[83]
PEDOT:PSS/HEC films	Sustainable sensor fabricated by coating	-	~100%	-	[89]

^1^ PDMS: polydimethylsiloxane; PAVK: polyacrylamide, polyvinyl pyrrolidone, and potassium chloride; PANS: polyacrylamide, poly(*N*-methylol) acrylamide, carboxymethyl chitosan, and calcium chloride; PANC: polyacrylamide, poly(*N*-methylol) acrylamide, carboxylated cellulose nano-whiskers; HEC: hydroxyethyl cellulose.

## Data Availability

No new data were created or analyzed in this study. Data sharing is not applicable to this article.
